# Hepatic microRNA expression is associated with the response to interferon treatment of chronic hepatitis C

**DOI:** 10.1186/1755-8794-3-48

**Published:** 2010-10-22

**Authors:** Yoshiki Murakami, Masami Tanaka, Hidenori Toyoda, Katsuyuki Hayashi, Masahiko Kuroda, Atsushi Tajima, Kunitada Shimotohno

**Affiliations:** 1 Center for Genomic Medicine, Kyoto University Graduate School of Medicine, 53 Shogoinkawahara-cho, Sakyo-ku, Kyoto 606-8507, Japan; 2 Department of Molecular Pathology, Tokyo Medical University, 6-1-1 Shinjuku, Shinjuku-ku, Tokyo 160-8402, Japan; 3 Department of Gastroenterology, Ogaki Municipal Hospital, 86-4 Minaminokawa-cho, Ogaki, Gifu 503-8502, Japan; 4 DNA Chip Research Inc., 43-1-1 Suehiro-cho, Tsurumi-ku, Yokohama, Kanagawa 230-0045, Japan; 5 Department of Molecular Life Science, Tokai University School of Medicine, 143 Shimokasuya, Isehara, Kanagawa 259-1193, Japan; 6 Research Institute, Chiba Institute for Technology, 2-17-1 Tsudanuma, Narashino, Chiba 275-0016, Japan

## Abstract

**Background:**

HCV infection frequently induces chronic liver diseases. The current standard treatment for chronic hepatitis (CH) C combines pegylated interferon (IFN) and ribavirin, and is less than ideal due to undesirable effects. MicroRNAs (miRNAs) are endogenous small non-coding RNAs that control gene expression by degrading or suppressing the translation of target mRNAs. In this study we administered the standard combination treatment to CHC patients. We then examined their miRNA expression profiles in order to identify the miRNAs that were associated with each patient's drug response.

**Methods:**

99 CHC patients with no anti-viral therapy history were enrolled. The expression level of 470 mature miRNAs found their biopsy specimen, obtained prior to the combination therapy, were quantified using microarray analysis. The miRNA expression pattern was classified based on the final virological response to the combination therapy. Monte Carlo Cross Validation (MCCV) was used to validate the outcome of the prediction based on the miRNA expression profile.

**Results:**

We found that the expression level of 9 miRNAs were significantly different in the sustained virological response (SVR) and non-responder (NR) groups. MCCV revealed an accuracy, sensitivity, and specificity of 70.5%, 76.5% and 63.3% in SVR and non-SVR and 70.0%, 67.5%, and 73.7% in relapse (R) and NR, respectively.

**Conclusions:**

The hepatic miRNA expression pattern that exists in CHC patients before combination therapy is associated with their therapeutic outcome. This information can be utilized as a novel biomarker to predict drug response and can also be applied to developing novel anti-viral therapy for CHC patients.

## Background

Hepatitis C virus (HCV) infection affects more than 3% of the world population. HCV infection frequently induces chronic liver diseases ranging from chronic hepatitis (CH) C, to liver cirrhosis (LC) and hepatocellular carcinoma (HCC) [1]. The current standard treatment for CHC combines pegylated interferon (Peg-IFN) and ribavirin, and has been found to be effective in only 50% of HCV genotype 1b infection. Furthermore this form of therapy is often accompanied by adverse effects; therefore, there is a pressing need to develop alternative strategies to treat CHC and to identify patients that will not be responsive to treatment [2].

MicroRNAs (miRNAs) are endogenous small non-coding RNAs that control gene expression by degrading or suppressing the translation of target mRNAs [3,4]. There are currently 940 identifiable human miRNAs (The miRBase Sequence Database -- Release 15.0). These miRNAs can recognize hundreds of target genes with incomplete complementary over one third of human genes appear to be conserved miRNA targets [5,6]. miRNA can associate not only several pathophysiologic events but also cell proliferation and differentiation.

However, there are many miRNAs whose functions are still unclear. Examples include miR-122 which is an abundant liver-specific miRNA that is said to constitute up to 70% of all miRNA molecules in hepatocytes [7]. The expression level of miR-122 was reportedly associated with early response to IFN treatment, while others like miR-26 have expression status that is associated with HCC survival and response to adjuvant therapy with IFN [8,9]. IFN beta (IFNβ) on the other hand, has been shown to rapidly modulate the expression of numerous cellular miRNAs, and it has been demonstrated that 8 IFNβ-induced miRNAs have sequence-predicted targets within the hepatitis C virus (HCV) genomic RNA [10]. Finally several miRNAs have been recognized as having target sites in the HCV genome that inhibits viral replication [10-12].

To date, various parameters have been examined in an attempt to confirm the effects of the IFN-related treatment for CHC. In patients with chronic HCV genotype 1b infection, there is a substantial correlation between responses to IFN and mutation in the interferon sensitivity determining region (ISDR) of the viral genome [13]. Substitutions of amino acid in the HCV core region (aa 70 and aa 91) were identified as predictors of early HCV-RNA negativity and several virological responses, including sustained response to standard combination therapy [14]. In order to assess the drug response to combination therapy for CHC using gene expression signatures, several researchers cataloged the IFN related gene expression profile from liver tissue or peripheral blood mononuclear cells (PBMC) [15,16]. It was found that failed combination therapy was associated with up-regulation of a specific set of IFN-responsive genes in the liver before treatment [17]. Additional reports have indicated that two SNPs near the gene IL28B on chromosome 19 may also be associated with a patient's lack of response to combination therapy [18]. These reports suggest that gene expression during the early phase of anti-HCV therapy may elucidate important molecular pathways for achieving virological response [19].

Our aim in this study was to identify gene related factors that contribute to poor treatment response to combination therapy for CHC. In order to achieve this we studied the miRNA expression profile of CHC patients before treatment with CHC combination therapy and tried to determine the miRNAs that were associated with their drug response. Knowing patients' expression profile is expected to provide a clearer understanding of how aberrant expression of miRNAs can contribute to the development of chronic liver disease as well as aid in the development of more effective and safer therapeutic strategies for CHC.

## Methods

### Patients and sample preparation

Ninety-nine CHC patients with HCV genotype 1b were enrolled (Table 1). Patients with autoimmune hepatitis, or alcohol-induced liver injury, or hepatitis B virus-associated antigen/antibody or anti-human immunodeficiency virus antibody were excluded. There were no patients who received IFN therapy or immunomodulatory therapy before enrollment in the study. Serum HCV RNA was quantified before IFN treatment using Amplicor-HCV Monitor Assay (Roche Molecular Diagnostics Co., Tokyo, Japan). Liver biopsy specimen was collected from each patient up to one week prior to administering combination therapy. Histological grading and staging of liver biopsy specimens from the CHC patients were performed according to the Metavir classification system. Pretreatment blood tests were conducted to determine each patient's level of aspartate aminotransferase, alanine aminotransferase, total bilirubin, alkaline phosphatase, gamma-glutamyl transpeptidase, white blood cell (WBC), platelets, and hemoglobin. Written informed consent was obtained from all of the patients or their guardians and provided to the Ethics Committee of the Graduate School of Kyoto University, who approved the conduct of this study in accordance with the Helsinki Declaration.

**Table 1:** Clinical characteristics of patients

Characteristics	SVR (n = 46)	R (n = 28)	NR (n = 25)	p-value
Age (years)	57.0 ± 9.8	61.2 ± 8.3	60.6 ± 7.6	0.09†

Male (%)	28 (61%)	11 (39%)	9 (36%)	0.08§

Weight (kg)	59.5 ± 9.0	56.6 ± 9.9	56.0 ± 7.7	0.13†

HCV RNA (×10^6 ^copies/ml)	1.90 ± 1.95	1.83 ± 1.04	1.58 ± 0.93	0.62†

Fibrosis stage				
	
F 0	1	1	1	0.50§
	
F 1	29	16	10	
	
F 2	10	7	6	
	
F 3	6	4	7	
	
F 4	0	0	1	

WBC(×10^3^/mm^3^)	5.31 ± 1.59	5.18 ± 1.24	4.71 ± 1.15	0.29†

Hemoglobin (g/dl)	14.2 ± 1.26	13.6 ± 1.35	13.5 ± 1.13	0.022†

Platelet (×10^4^/mm^3^)	16.7 ± 5.0	16.4 ± 4.0	15.2 ± 6.1	0.25†

AST (IU/L)	54.8 ± 48.1	46.6 ± 29.3	57.0 ± 28.5	0.17†

ALT (IU/L)	74.5 ± 87.8	47.9 ± 28.6	67.6 ± 43.2	0.15†

γGTP (IU/L)	56.0 ± 69.4	38.5 ± 28.9	74.3 ± 59.0	0.025†

ALP (IU/L)	248 ± 71.5	245 ± 75.7	323 ± 151	0.038†

Total Bilirubin (mg/dl)	0.67 ± 0.22	0.72 ± 0.30	0.68 ± 0.19	0.95†

Albumin (g/dl)	4.21 ± 0.31	4.13 ± 0.27	4.01 ± 0.48	0.14†

Abbreviations. SVR, sustained virological response; R, relapse; NR, non responder; Differences in clinical characteristics among three groups were tested using †Kruskal-Wallis test, or §Fisher's exact test. AST, aspartate aminotransferase; ALT, alanine aminotransferase; WBC, white blood cell; ALP, alkaline phosphatase; γGTP, gamma-glutamyl transpeptidase.

### Treatment protocol and definitions

**Figure 1:**
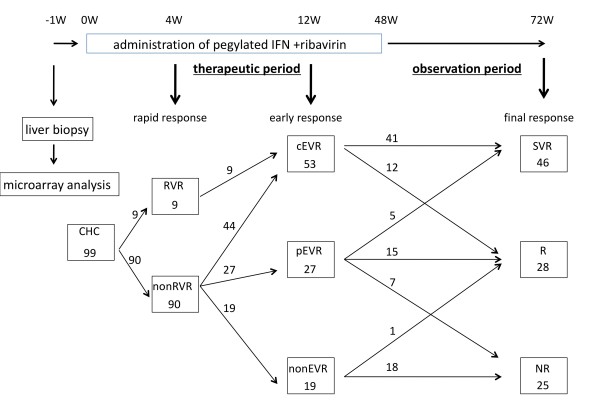
**Study design and time-line response to combination therapy**. The time-course of liver biopsy, microarray analysis, therapeutic period, observation period after combination therapy, and drug response judgment time (4W, 12W, 72W) is shown. Each therapeutic result (rapid, early, and final response) and the number of patients transitioning into each group are shown. RVR, cEVR, pEVR, SVR, R, and NR are denoted as rapid virological response, complete early virological response, partial EVR, sustained virological response, relapse, and non responder, respectively.

All enrolled patients were treated with pegylated IFNa-2b (Schering-Plough Corporation, Kenilworth, NJ, USA) and ribavirin (Schering-Plough) for 48 weeks (Figure 1). Pegylated IFN was administered at a dose of 1.5 mg kg/week at the starting point. Ribavirin was administered following the dose recommended by the manufacturer.

### Definitions of drug response to therapy

Drug response was defined according to how much HCV RNA had decreased in each patient's serum. After four weeks of drug administration (rapid response phase) the patients were classified into the following two groups after four weeks of drug administration: (i) rapid virological responder (RVR): a patient whose serum was negative for serum HCV RNA at four weeks, and (ii) non-RVR: a patient who was not classified as RVR.

The patients were classified into the following three groups after 12 weeks of drug administration (early response phase): (i) complete early virological responder (cEVR): a patient who was negative for serum HCV RNA at 12 weeks; (ii) partial EVR: a patient whose serum HCV RNA was reduced by 2-log or more of the HCV RNA before drug administration at 12 weeks, but who was not negative for serum HCV RNA; and (iii) non-EVR: a patient who was not classified as either cEVR or pEVR.

The patients were classified into the following three groups at the time of post-treatment at 24 weeks (final response): (i) sustained virological responder (SVR): a patient who was negative for serum HCV RNA during the six months following completion of the combination therapy; (ii) relapse (R): a patient whose serum HCV RNA was negative by the end of the combination therapy but reappeared after completion of the combination therapy; and (iii) virological non-responder (NR): a patient who was positive for serum HCV RNA during the entire course of the combination treatment.

### RNA extraction

Liver biopsy specimens were stored in RNA later (Ambion, Austin, TX, USA) at -80°C until RNA extraction. Total RNA was extracted by using mirVana™ miRNA Isolation kit (Ambion) according to the manufacturer's instruction.

### miRNA microarray

miRNA microarrays were manufactured by using Agilent Technologies (Santa Clara, CA, USA). Total RNA (100 ng) were labeled and hybridized using a Human microRNA Microarray kit (Agilent Technologies) according to the manufacturer's protocol (Protocol for use with Agilent microRNA microarrays Version 1.5). Hybridization signals were detected using the DNA microarray scanner G2505B (Agilent Technologies), and all scanned images were analyzed using Agilent feature extraction software (v9.5.3.1). Data were analyzed using GeneSpring GX 7.3.1 software (Agilent Technologies) and normalized as follows: (i) values below 0.01 were set to 0.01. (ii) each measurement was divided by the 75th percentile of all measurements to compare one-color expression profiles. The data presented in this manuscript have been deposited in NCBI's Gene Expression Omnibus and are accessible through GEO Series accession number GSE16922: http://www.ncbi.nlm.nih.gov/geo/query/acc.cgi?token=xlmbxyyumcwkeba&acc=GSE16922

### Real-time qPCR for miRNA quantification

To detect the expression level of miRNA by real-time qPCR, TaqMan^® ^microRNA assay (Applied Biosystems) was used to quantify the relative expression levels of miR-18a (assay ID, 002422), miR-27b (assay ID, 000409), miR-422b (assay ID, 000575), miR-143 (assay ID, 000466), miR-145 (assay ID, 000467), miR-34b (assay ID, 000427), miR-378 (assay ID, 000567) and U18 (assay ID, 001204) which was used as an internal control. cDNA was synthesized by Taqman miRNA RT Kit (Applied Biosystems). Total RNA (10 ng) in 5 ul of nuclease free water was added to 3 ul of 5× RT primer, 10× 1.5 ul of reverse transcriptase buffer, 0.15 ul of 100 mM dNTP, 0.19 ul of RNase inhibitor, 4.16 ul of nuclease free water, and 50 U of reverse transcriptase in a total volume of 15 ul. The reaction was performed for 30 min at 16°C, 30 min at 42°C, and five min at 85°C. All RT reactions were run in triplicate. Chromo 4 detector (BIO-RAD, Hercules, CA, USA) was used to detect miRNA expression.

### Method of predicting prognosis

Monte Carlo Cross Validation (MCCV) was used to identify a set of prognostic miRNAs and to assess and predict drug response [20,21]. We chose MCCV to make up for relatively small number of patients. The 99 enrolled patients were repeatedly and randomly divided 100 times into training sets (TSs; size n = 10, 20, ..., 90) and a corresponding validation set (VS; size = 99-n). The percentile-normalized measures for miRNA expression were compared between the 2 TS patient groups of SVR and non-SVR (R and NR) by computing absolute values of the difference for each of the 172 miRNAs that were higher than 10. A prognosis signature was defined in terms of the expression measures of the miRNAs with the largest absolute differences. A 35-miRNA prognosis predictor (PP) was established for TS patients and its performance was assessed on VS patients. A PP was computed by applying diagonal linear discriminant analysis to the 35-miRNA PP of the TS patients (Table 2 and 3). The PP was applied to predict the prognoses of the VS patients. The predicted and actual prognoses (SVR or non-SVR) of the VS patients were compared to obtain the following three measures of prognosis prediction performance: (1) accuracy (proportion of correctly predicted prognoses), (2) sensitivity (proportion of correctly predicted non-SVR) and (3) specificity (proportion of correctly predicted SVR). 53 patients (N and NR) were also repeatedly and randomly divided 100 times into training sets (TSs; size n = 6, 12, ...42) and corresponding validation set (VS; size = 53-n). Perl programs of our own writing performed all analytical processes.

**Table 2:** List of the 35 miRNAs used to classify patients into SVR and non-SVR groups using Monte Carlo Cross Validation (MCCV)

Gene name	fold change (SVR/non SVR)	T-test		Selection by MCCV	
			**Rank**	**appearance frequency in this classification (%)**	**appearance number of times**

hsa-miR-122a	1.32	6.67E-02	1	98.78	889
hsa-miR-21	1.19	3.62E-01	2	94.67	852
hsa-miR-22	1.23	7.80E-02	3	93.22	839
hsa-let-7a	1.14	3.57E-01	4	92.33	831
hsa-miR-23b	1.41	1.72E-02	5	91.44	823
hsa-miR-26a	1.32	7.45E-02	6	90.78	817
hsa-let-7f	1.15	4.04E-01	7	88.67	798
hsa-miR-142-3p	1.39	1.45E-01	8	87.33	786
hsa-miR-494	2.18	5.85E-03	9	82.00	738
hsa-miR-194	1.22	1.70E-01	10	80.78	727
hsa-let-7b	1.11	3.59E-01	11	80.22	722
hsa-miR-148a	1.25	2.28E-01	12	79.67	717
hsa-miR-29a	1.16	2.73E-01	13	77.78	700
hsa-miR-125b	1.20	2.37E-01	14	73.11	658
hsa-miR-192	1.09	4.89E-01	15	69.67	627
hsa-miR-24	1.25	8.31E-02	16	68.89	620
hsa-miR-768-3p	1.19	1.78E-01	17	68.78	619
hsa-miR-126	1.07	6.75E-01	18	49.56	446
hsa-miR-19b	1.15	2.98E-01	19	48.89	440
hsa-miR-370	2.00	1.44E-02	20	39.00	351
hsa-miR-29c	1.26	1.37E-01	21	38.89	350
hsa-miR-16	1.24	2.08E-01	22	37.11	334
hsa-miR-145	1.01	9.25E-01	23	34.89	314
hsa-let-7c	1.21	1.41E-01	24	33.22	299
hsa-miR-215	1.20	3.65E-01	25	27.67	249
hsa-let-7g	1.16	3.64E-01	26	27.44	247
hsa-miR-451	1.13	6.94E-01	27	23.11	208
hsa-miR-26b	1.30	2.26E-01	28	22.22	200
hsa-miR-92	1.12	3.44E-01	29	21.11	190
hsa-miR-29b	1.19	2.62E-01	30	19.44	175
hsa-miR-107	1.21	1.58E-01	31	18.78	169
hsa-miR-27b	1.40	2.32E-02	32	18.11	163
hsa-miR-638	1.32	5.57E-02	33	16.89	152
hsa-miR-199a*	1.12	5.92E-01	34	16.78	151
hsa-miR-193b	1.25	7.24E-02	35	16.67	150

**Table 3:** List of the miRNAs used to classify patients into R and NR groups

Gene name	fold change (R/NR)	T-test		Selection by MCCV	
			**Rank**	**appearance frequency in this classification (%)**	**appearance number of times**

hsa-miR-122a	1.50	6.70E-02	1	98.57	690
hsa-miR-21	1.13	5.43E-01	2	89.86	629
hsa-let-7a	1.15	4.23E-01	3	88.71	621
hsa-let-7f	1.24	3.01E-01	4	87.43	612
hsa-miR-148a	1.70	4.51E-02	5	82.71	579
hsa-miR-192	1.24	1.93E-01	6	81.71	572
hsa-miR-126	1.21	3.19E-01	7	74.14	519
hsa-miR-22	1.04	7.88E-01	8	68.43	479
hsa-miR-194	1.20	3.63E-01	9	64.29	450
hsa-miR-23b	1.30	2.06E-01	10	62.00	434
hsa-miR-125b	1.23	2.88E-01	11	61.86	433
hsa-miR-494	0.45	8.17E-02	12	61.14	428
hsa-miR-19b	1.17	3.86E-01	13	61.14	428
hsa-miR-29a	1.11	5.44E-01	14	59.86	419
hsa-miR-26a	1.13	5.38E-01	15	58.43	409
hsa-let-7b	1.01	9.37E-01	16	56.86	398
hsa-miR-142-3p	1.15	5.54E-01	17	52.71	369
hsa-miR-215	1.28	3.93E-01	18	52.00	364
hsa-miR-101	1.31	1.26E-01	19	49.00	343
hsa-miR-451	1.35	5.25E-01	20	48.14	337
hsa-miR-145	0.99	9.76E-01	21	47.14	330
hsa-let-7g	1.15	4.84E-01	22	44.00	308
hsa-miR-29c	1.23	2.94E-01	23	43.71	306
hsa-miR-26b	1.37	2.85E-01	24	43.14	302
hsa-miR-768-3p	1.00	9.91E-01	25	36.29	254
hsa-let-7c	1.16	3.76E-01	26	36.14	253
hsa-miR-370	0.43	7.36E-02	27	35.57	249
hsa-miR-92	1.07	6.65E-01	28	34.14	239
hsa-miR-16	1.11	6.18E-01	29	26.71	187
hsa-miR-29b	1.14	5.19E-01	30	25.71	180
hsa-miR-27b	1.40	1.15E-01	31	25.71	180
hsa-miR-24	1.08	6.56E-01	32	20.57	144
hsa-miR-107	1.00	9.81E-01	33	19.57	137
hsa-miR-143	0.95	7.99E-01	34	18.43	129
hsa-miR-214	0.85	3.61E-01	35	17.86	125

### Cell lines and miRNA transfection

HEK293 cells were maintained in D-MEM (Invitrogen, Carlsbad, CA, USA) with 10% fetal bovine serum, plated in 60 mm diameter dishes and cultured to 70% confluence. 293 cells were plated in 6-well plates the day before transfection and grown to 70% confluence. Cells were transfected with 50 pmol of Silencer^® ^negative control siRNA (Ambion) or double-stranded mature miRNA (ds miRNA) or 2'-O-methylated antisense oligonucleotide against the miRNA of interest (ASO miRNA) (Hokkaido System Science, Sapporo, Japan) using lipofectamine RNAiMAX (Invitrogen). Cells were harvested 2 days after transfection.

### Real-time qPCR for mRNA quanification

cDNA was synthesized using the Transcriptor High Fiderity cDNA synthesis Kit (Roche, Basel, Switzerland). Total RNA (2 μg) in 10.4 μl of nuclease free water was added to 1 μl of 50 mM random hexamer. The denaturing reaction was performed for 10 min at 65°C. The denatured RNA mixture was added to 4 μl of 5× reverse transcriptase buffer, 2 μl of 10 mM dNTP, 0.5 μl of 40 U/μl RNase inhibitor, and 1.1 μl of reverse transcriptase (FastStart Universal SYBR Green Master (Roche) in a total volume of 20 μl. The reaction ran for 30 min at 50°C (cDNA synthesis), and five min at 85°C (enzyme denaturation). All reactions were run in triplicate. Chromo 4 detector (BIO-RAD, Hercules, CA, USA) was used to detect mRNA expression. The primer sequences are as follows; BCL2 s; 5'-gttgctttacgtggcctgtt-3', as; 5'-ggaggtctggcttcatacca-3', RARA s; 5'-cataccctgccataccaacc-3', as; 5'-gacatgaaaggagagtgggg-3', SMAD2 s; 5'-aatattttggggactgatgcc-3', as; 5'-gcttttgggcagtggttaag-3', and β-actin s; 5'-ccactggcatcgtgatggac-3', as; 5'-tcattgccaatggtgatgacct-3'. Assays were performed in triplicate, and the expression levels of target genes were normalized to the expression of the β-actin gene, as quantified using real-time qPCR as internal controls.

### Statistical analysis

Data were statistically analyzed using the Student's t-test and differences in clinical characteristics among 3 groups were tested using the Kruskal-Wallis test, or Fisher's exact test. Data from microarray were also statically analyzed using Welch's test and Benjamini-Hochberg correction for multiple hypotheses testing.

## Results

A microarray platform was used to determine miRNA expression of 470 miRNAs in 99 fresh-frozen CHC liver tissues.

### miRNAs which related to the final response of combination therapy

**Figure 2:**
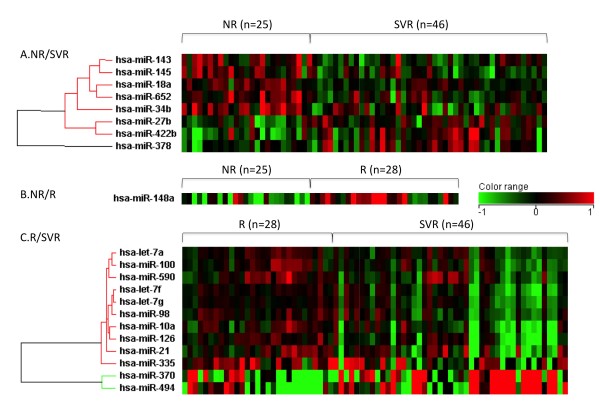
**Clustering of CHC patients according to their final response to Peg-IFN and ribavirin combination therapy**. Heatmap using miRNA differently expressed among SVR, R, and NR is shown (A: classification between NR and SVR, B: classification between NR and R, and C: classification between R and SVR, respectively). Vertical bars represent the miRNA genes and the horizontal bars represent the samples. Green bars reflect downregulated genes and red bars upregulated genes.

Unique miRNA expression patterns were established according to the final virological response (SVR, R, and NR) to the combination therapy (Figure 1). To isolate the miRNAs that were associated with the drug response to the combination therapy, we chose miRNAs which had ≥ 1.25 fold difference in the mean values of the gene expression level between at least two groups (p < 0.05). Unsupervised hierarchical clustering based on all the miRNAs spotted on the chip, revealed a marked, very distinct separation according to the patients' final response of the CHC liver tissue to the Peg-IFN and ribavirin combination therapy (Figure 2).

The result was that the expression level of 3 miRNAs (miR-27b, miR-378, miR-422b) in SVR was significantly higher than that in NR, whereas the expression level of 5 miRNAs (miR-34b, miR-145, miR-143, miR-652, and miR-18a) in SVR was significantly lower than that in NR. Without FDR correction, the expression level of miR-122 in NR was lower than that in SVR. The expression level of 2 miRNAs in SVR was significantly higher than that in R, whereas the expression level of 10 miRNAs in SVR was significantly lower than in that in R. Additionally, the expression level of miR-148a in R was significantly higher than that in NR. There was no significant difference in the expression level of miR-122 in NR and R (Table 4).

**Table 4:** Extracted miRNA related to the final outcome of combination therapy

Gene Name	Fold Change (NR/SVR)	p-value with FDR correction	p-value without correction
hsa-miR-34b*	1.50	3.53E-02	6.95E-05
hsa-miR-145	1.35	3.55E-02	5.50E-05
hsa-miR-143	1.31	4.65E-02	6.46E-04
hsa-miR-652	1.28	4.33E-02	3.43E-04
hsa-miR-18a	1.22	4.33E-02	2.02E-05
hsa-miR-27b	0.78	4.33E-02	3.97E-05
hsa-miR-422b*	0.71	4.33E-02	1.44E-04
hsa-miR-378	0.70	4.86E-02	1.38E-03
hsa-miR-122	0.72	> 5.00E-02	2.59E-04
			

**Gene Name**	**Fold Change (NR/R)**	**p-value with FDR correction**	**p-value without correction**

hsa-miR-148a	0.59	1.60E-02	8.99E-04
has-miR-122	0.72	> 5.00E-02	6.23E-04
			

**Gene Name**	**Fold Change (R/SVR)**	**P-value**	**p-value without correction**

hsa-let-7a	1.15	3.93E-02	1.94E-03
hsa-let-7f	1.24	1.04E-02	3.60E-03
hsa-let-7g	1.17	1.93E-02	1.82E-02
hsa-miR-100	1.23	1.93E-02	9.23E-04
hsa-miR-10a	1.37	1.26E-02	2.40E-03
hsa-miR-126	1.36	1.04E-02	1.50E-03
hsa-miR-21	1.30	4.78E-02	3.45E-02
hsa-miR-335	2.00	2.83E-02	3.50E-02
hsa-miR-370	0.36	1.38E-02	2.96E-03
hsa-miR-494	0.37	3.93E-02	1.97E-03
hsa-miR-590	1.26	3.93E-02	5.59E-03
hsa-miR-98	1.22	1.38E-02	6.64E-03

Asterisk was denoted the common miRNAs appeared whose expression level between NR and SVR, and nonEVR and cEVR.

### Validation of the microarray result by real-time qPCR

**Figure 3:**
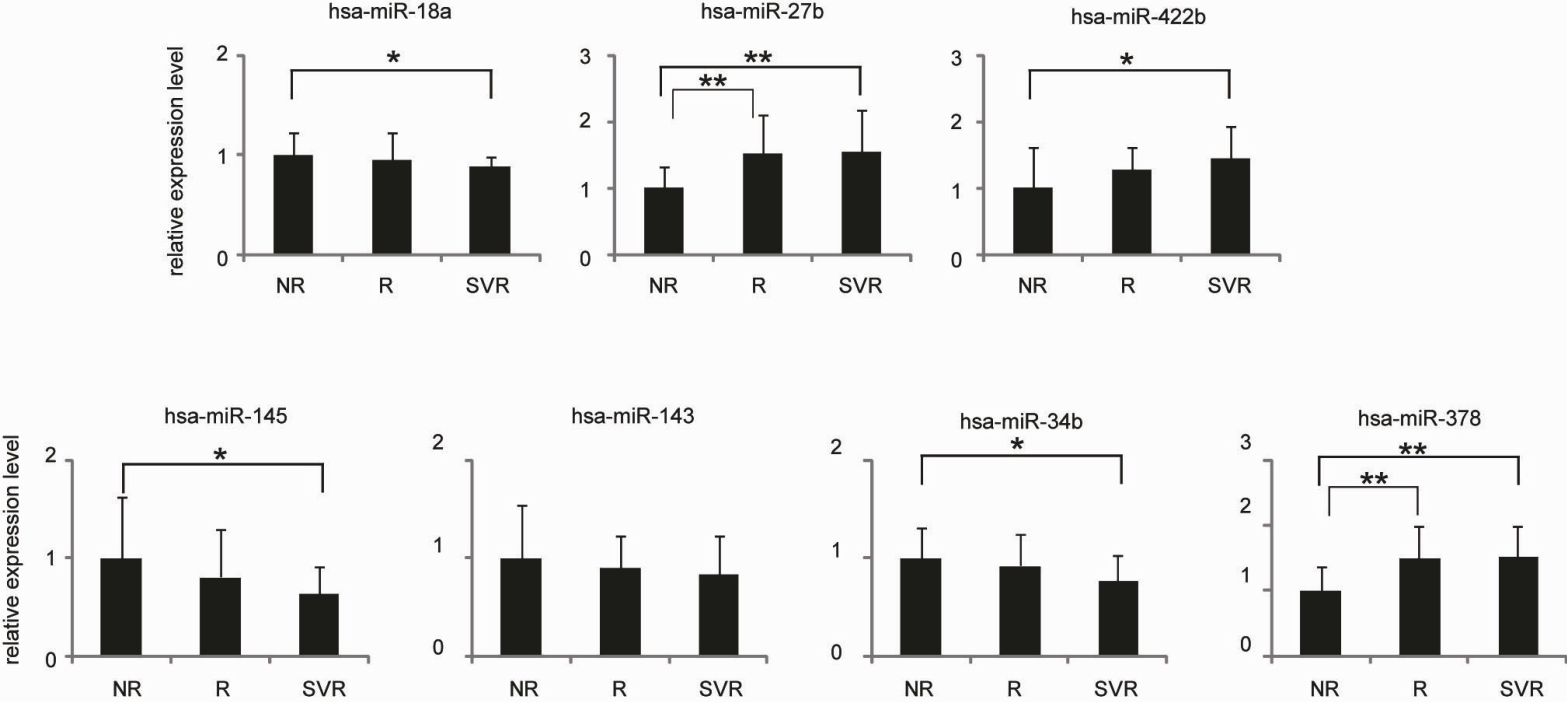
**Real-time qPCR validation of the seven miRNAs**. Each column represents the relative amount of miRNAs normalized to expression level of U18. The data shown were means+SD of three independent experiments. Asterisk indicates a significant difference of p < 0.05 (*) or p < 0.01 (**).

The three miRNAs (miR-18a, miR-27b, and miR-422b) with the smallest difference of fold change between NR and SVR groups and four miRNA (miR-143, miR-145, miR-34b, and miR-378) with the largest difference of fold change between NR and SVR groups were chosen to confirm the microarray results using stem-loop based real-time qPCR. The result of real-time qPCR corresponded to the result from the microarray analysis (Figure 3).

### miRNAs which related to the 4 week (rapid response phase) response to combination therapy

The miRNA expression profile was established according to the rapid phase response to the combination therapy by week 4 (Table 5). Our results showed that the expression level of 5 miRNAs in non-RVR was significantly higher than that in RVR. Prior results have revealed that a patient who achieves RVR as a result of the combination therapy has a high possibility of achieving SVR [22,23]. Our research supports this finding: nine out of 99 patients achieved RVR. All nine cases shifted to cEVR by week 12, and 8 shifted to SVR at the final response. The 90 cases in non-RVR shifted to 44 cases in cEVR, 19 in pEVR, and 27 in non-EVR and at the final response shifted to 38 cases in SVR, 27 in R, and 25 in NR (Table 6 and Figure 1).

**Table 5:** List of the miRNA related to the rapid or early outcome of combination therapy

Gene Name	Fold Change (non RVR/RVR)	p-value	p-value without correction
hsa-let-7c	1.17	2.01E-02	8.31E-03
hsa-let-7d	1.13	3.50E-02	5.63E-02
hsa-miR-139	1.29	3.35E-02	2.70E-02
hsa-miR-324-5p	1.14	1.64E-02	3.24E-02
hsa-miR-768-5p	1.34	4.57E-02	1.29E-02

			

**Gene Name**	**Fold Change (non EVR/cEVR)**	**p-value**	**p-value without correction**

hsa-miR-34b*	1.51	3.30E-02	1.69E-04
hsa-miR-23b	0.74	2.69E-02	8.91E-05
hsa-miR-422b*	0.67	2.40E-02	1.34E-04
			
hsa-miR-122	0.74	> 5.00E-02	3.07E-03

Asterisk was denoted the common miRNAs appeared whose expression level between NR and SVR, and nonEVR and cEVR.

**Table 6:** Patients' periodical drug response changes

**code No**.	4W treatment (rapid response)	12W treatment (early response)	48W treatment +24W observation (final outcome)	code No	4W treatment (rapid response)	12W treatment (early response)	48W treatment +24W observation (final outcome)
OCH-105	non RVR	non EVR	NR	OCH-103	RVR	cEVR	SVR
OCH-111	non RVR	pEVR	NR	OCH-104	non RVR	cEVR	SVR
OCH-118	non RVR	non EVR	NR	OCH-107	non RVR	cEVR	SVR
OCH-119	non RVR	non EVR	NR	OCH-108	non RVR	cEVR	SVR
OCH-122	non RVR	pEVR	NR	OCH-109	non RVR	cEVR	SVR
OCH-123	non RVR	non EVR	NR	OCH-110	non RVR	cEVR	SVR
OCH-126	non RVR	non EVR	NR	OCH-112	non RVR	pEVR	SVR
OCH-127	non RVR	non EVR	NR	OCH-114	RVR	cEVR	SVR
OCH-132	non RVR	pEVR	NR	OCH-116	non RVR	cEVR	SVR
OCH-137	non RVR	non EVR	NR	OCH-121	non RVR	pEVR	SVR
OCH-140	non RVR	pEVR	NR	OCH-124	non RVR	cEVR	SVR
OCH-142	non RVR	non EVR	NR	OCH-130	non RVR	cEVR	SVR
OCH-144	non RVR	pEVR	NR	OCH-131	non RVR	cEVR	SVR
OCH-145	non RVR	non EVR	NR	OCH-136	non RVR	cEVR	SVR
OCH-192	non RVR	non EVR	NR	OCH-138	non RVR	cEVR	SVR
OCH-204	non RVR	non EVR	NR	OCH-139	non RVR	cEVR	SVR
OCH-205	non RVR	non EVR	NR	OCH-143	non RVR	cEVR	SVR
OCH-206	non RVR	non EVR	NR	OCH-150	non RVR	cEVR	SVR
OCH-207	non RVR	non EVR	NR	OCH-153	non RVR	cEVR	SVR
OCH-208	non RVR	non EVR	NR	OCH-154	non RVR	cEVR	SVR
OCH-209	non RVR	pEVR	NR	OCH-155	non RVR	cEVR	SVR
OCH-210	non RVR	non EVR	NR	OCH-156	non RVR	cEVR	SVR
OCH-211	non RVR	non EVR	NR	OCH-157	non RVR	pEVR	SVR
OCH-223	non RVR	non EVR	NR	OCH-158	non RVR	pEVR	SVR
OCH-242	non RVR	pEVR	NR	OCH-160	non RVR	cEVR	SVR
OCH-101	non RVR	cEVR	R	OCH-186	non RVR	cEVR	SVR
OCH-102	RVR	cEVR	R	OCH-187	non RVR	cEVR	SVR
OCH-106	non RVR	non EVR	R	OCH-189	non RVR	pEVR	SVR
OCH-113	non RVR	pEVR	R	OCH-190	non RVR	cEVR	SVR
OCH-115	non RVR	cEVR	R	OCH-191	RVR	cEVR	SVR
OCH-117	non RVR	cEVR	R	OCH-194	non RVR	cEVR	SVR
OCH-120	non RVR	pEVR	R	OCH-195	non RVR	cEVR	SVR
OCH-125	non RVR	pEVR	R	OCH-222	non RVR	cEVR	SVR
OCH-128	non RVR	pEVR	R	OCH-228	non RVR	cEVR	SVR
OCH-129	non RVR	pEVR	R	OCH-229	RVR	cEVR	SVR
OCH-133	non RVR	pEVR	R	OCH-230	non RVR	cEVR	SVR
OCH-134	non RVR	pEVR	R	OCH-231	non RVR	cEVR	SVR
OCH-135	non RVR	cEVR	R	OCH-232	non RVR	cEVR	SVR
OCH-141	non RVR	cEVR	R	OCH-233	non RVR	cEVR	SVR
OCH-151	non RVR	pEVR	R	OCH-234	RVR	cEVR	SVR
OCH-152	non RVR	pEVR	R	OCH-236	non RVR	cEVR	SVR
OCH-159	non RVR	pEVR	R	OCH-237	non RVR	cEVR	SVR
OCH-188	non RVR	cEVR	R	OCH-238	non RVR	cEVR	SVR
OCH-213	non RVR	cEVR	R	OCH-240	RVR	cEVR	SVR
OCH-214	non RVR	pEVR	R	OCH-241	RVR	cEVR	SVR
OCH-215	non RVR	pEVR	R	OCH-243	RVR	cEVR	SVR
OCH-216	non RVR	cEVR	R				
OCH-217	non RVR	pEVR	R				
OCH-218	non RVR	cEVR	R				
OCH-219	non RVR	pEVR	R				
OCH-220	non RVR	cEVR	R				
OCH-221	non RVR	pEVR	R				
OCH-239	non RVR	cEVR	R				

### miRNAs which related to the 12 week (early response phase) response to combination therapy

Establishing the miRNA expression profile of patients according to their 12 week (early response) of CHC liver specimen to the combination therapy after 12 weeks, showed that the expression level of miR-23b and miR-422b in cEVR was higher than that in non-EVR, and the expression level of miR-34b in cEVR lower than that in non-EVR (Table 5). There were no miRNAs with expression level that differed significantly between cEVR and pEVR, and non-EVR and pEVR. The drug response at 12 weeks appeared to be a predictive factor of the final drug response. The 53 cases in cEVR at week 12 shifted to 41 cases in SVR and 12 in R at the final response. 27 cases in pEVR at week 12, shifted to 5 in SVR, 15 in R, and 7 in NR and 19 in non-EVR shifted to 1 in R and 18 in NR (Table 6 and Figure 1).

### Predicting the final outcome before drug administration using MCCV

**Figure 4:**
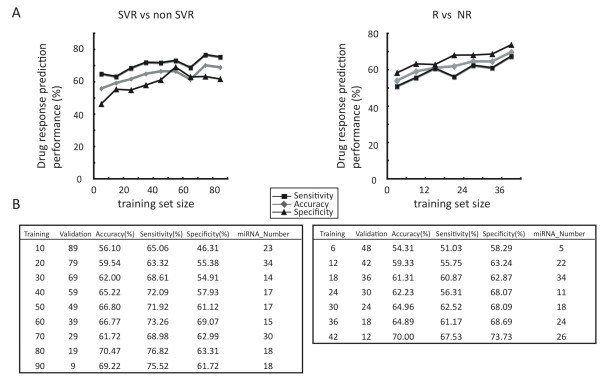
**Method for predicting the clinical outcome using MCCV**. Prediction performance of signatures from 35 miRNAs (Table 2 and 3). Left: Mean accuracy, specificity, and sensitivity (% in vertical axis) as a function of the training set size were determined for the 100 random splits of patients (non-SVR (NR+R) vs. SVR). Right: prediction performance of the training set size was determined by performing 48 random splits of patients (NR vs. R). A. Prediction performance (mean accuracy, specificity, and sensitivity) and number of miRNAs which was used for the prediction are also shown in each training set (TS) and validation set (VS).

Before initial drug administration, we attempted to simulate the clinical outcome of the combination therapy before drug administration by using MCCV. We first extracted the SVR and non-SVR groups from all of the patients, and then the R and NR groups were predicted afterwards. MCCV simulation showed that the accuracy, specificity, and sensitivity of the liver specimen classified as SVR or non-SVR was up to 70.5%, 63.3%, and 76.8%, respectively (TSs = 80). On the other hand, the accuracy, specificity, and sensitivity of the liver specimen classified as R or NR was 70.0%, 73.7%, and 67.5%, respectively (TSs = 42)(Figure 4). Fold change of their normalized expression level, P value, and number of selection by MCCV in the 35 informative miRNAs that were identified based on the all patients are shown in Table 2 and 3.

### miRNAs related to the final drug response can regulate the immune related genes

In order to clarify the biological links between miRNAs and IFN responses, we examined whether the expression of immune-related hypothetical miRNAs target genes (additional file 1) could be controlled by miRNAs which were related to the final drug response. We observed the changes in expression level of B-cell CLL/lymphoma 2 (BCL2), retinoic acid receptor, alpha (RARA), and SMAD family member 2 (SMAD2) by real-time qRT-PCR as the expression level of miRNAs (miR-143, miR-27b, and miR-18a) was modified, respectively, in HEK293 cells. The expression level of the hypothetical targets examined was down-regulated by over-expression of the corresponding miRNA and the corresponding antisense oligonucleotide (ASO) inhibited the function of miRNA (additional file 2).

## Discussion

Our large and comprehensive screening revealed that hepatic miRNA expression can be associated with a patient's drug response. There are several reports that miRNAs are closely related to innate immunity, and in this study, we found that several miRNAs had the potential to recognize immuno-related genes as target candidates [24-26]. For example, the following hypothetical candidate genes of miR-378, miR-18a, miR-27b, miR-34b, and miR-145 each identified as target genes, Interferon Response Factor (IRF) 1, IRF2, IRF4, IRF6, and IRF7, respectively (additional file 1). Past reports show that miR-422b was related to the B cell differentiation [27]. When an immuno-reaction induces aberrant expression of miRNA, the expression level of miR-34b significantly decreased in H69 cells following IFN-γ stimulation [28]. Bcl-6 positively directs follicular helper T cell differentiation, through combined repression of miR-18a and miR-27b and transcription factors [29].

In our study, there was significant difference in the fold change of the expression level of miRNA based on the drug response, however, the absolute value of the fold change was not so significant (Table 4). Usually one miRNA can regulate many genes including immuno-related gene (additional file 1), and these genes in turn can synergistically affect immune activity. In our preliminary study (additional file 2) BCL-2, RARA, and SMAD2 can be regulated by miR-143, miR-18a, and miR-27b, respectively. Considering that the expression level of several miRNAs changed these minute changes taken together can have a significant impact on a patient's drug-response and innate immunity.

Aberrant expression of miRNA can modify the replication of HCV. According to Vita algorithm, several miRNAs, related to drug response, can recognize HCV genotype 1b sequence as a target (additional file 3) [30]. For example, miR-199a* is able to target the HCV genome and inhibit viral replication [12]. IFN has the ability to modulate expression of certain miRNAs that may either target the HCV RNA genome (miR-196 or miR-448) or markedly enhance its replication (miR-122) [10,11]. The low expression level of miR-122 in the subjects shown in the NR group is in accordance with our results, however, after miRNA expression profile with FDR correction, the expression level of miR-122 was not significantly different between SVR and NR groups [8]. One reason why this difference is that their study comprised of patients infected with HCV genotype from 1 to 4 while this study consisted of HCV genotype 1b patients only.

The expression pattern of mRNA in HCV infected liver tissue is different from that of healthy tissue [15]. The expression pattern of the IFN-related genes in liver tissue before administering of IFN therapy, also differs according to the drug response [15,19]. The amount of plasmacytoid dendritic cell (pDC), which are the most potent secretors of antiviral Type-I IFN, has been shown to decrease in the peripheral blood of patients, however, pDC tend to become trapped in the liver tissue if HCV infection is present [31,32]. Taken together, it is possible that the variation in the miRNA expression pattern according to the drug response existed even before therapy.

Previously, large randomized controlled trials of IFN therapy for CHC, identified at pre-treatment stage several possible factors which are associated with the final virological response. These factors include: genotype, amount of HCV RNA in peripheral blood, degree of fibrosis, age, body weight, ethnicity, and steatosis [33]. Viral genome mutation in the ISDR region and the substitutions of amino acid in the HCV core region also served as predictors of early, as well as end-of treatment response [13,14]. The miRNA expression obtained from the therapeutic response, can be applied to the prediction of drug response. The advantages of using miRNA for the microarray analysis include the following; (i) It was relatively easy to analyze because fewer probes were installed compared with the usual cDNA array. (ii) The change in each manifestation of a miRNA was low, in fact, in most miRNA, standard deviation was twice or less in average value (data not shown). The expression levels of miR-34b and 422b in the early response phase and final responses to treatment were consistently and significantly high and low in non-responders, respectively. Therefore these two miRNAs may be useful markers for early-to-final drug response to the IFN treatment.

Further studies are indeed needed to clarify the connection between miRNA expression and patient response to CHC combination therapy. Because information on miRNA is regularly being updated, we are planning to performed more analysis using the latest microarray and a larger sample in the future. However, in the meantime, as we have shown in Figure 4, the bigger the size of the training set, the higher the prediction performance that is achieved. This combined with the results of our Monte Carlo cross validation provided a strong based to verify the concepts in this report. We believe that our results have three advantages (i) the prediction methods used were quite reasonable, (ii) the prediction performance can later be improved if more patients' data become available and (iii) obtaining miRNA profile (not specific miRNAs) is useful for predicting the drug response. While current therapy is based on positive selection with HCV genotype or negative selection with IL28B SNP, and is limited to only some cases, our methods are applicable to all patients [13,18].

## Conclusions

Our study shows that the specific miRNA are expressed differently depending on patient's drug response. As result we feel that miRNA profiling can be useful for predicting patient drug response before the administering combination therapy thereby reducing ineffective treatments. Moreover, miRNA expression profile can facilitate the accumulation of basal information for the development of novel therapeutic strategies. This approach allows for more suitable therapeutic strategies based on clinical information of individuals.

## List of abbreviations

HCV: hepatitis C virus; CH: chronic hepatitis C; LC: liver cirrhosis; HCC: hepatocellular carcinoma; miRNA: microRNA; IFN, interferon; SVR: sustained virological responder; R: relapse; NR: non-responder; RVR: rapid virological responder; EVR: early virological responder.

## Competing interests

The authors declare that they have no competing interests.

## Authors' contributions

YM and KS conceived and designed the experiments; YM, HT and KH performed the experiments; MT and MK performed statistical analysis; YM, MT, HT and AT contributed to writing and editing the manuscript. All authors read and approved the manuscript.

## Pre-publication history

The pre-publication history for this paper can be accessed here:

http://www.biomedcentral.com/1755-8794/3/48/prepub

## Supplementary Material

Additional file 1 **miRNA hypothetical target genes according to in silico analysis**. Click here for file

Additional file 2 **Real-time qPCR validation of immune-related hypothetical target genes of miRNAs**. The expression levels of hypothetical target genes in HEK293 cells were compared among three groups treated with control RNA, ds miRNA, and ASO miRNA. The data shown are means+SD of three independent experiments. Asterisk indicates a significant difference of p < 0.05. Click here for file

Additional file 3 human miRNA target on the HCV genome genotype 1b (Accession No. AF333324) Click here for file
