# Supplementary material for: Hepatic microRNA expression is associated with the response to interferon treatment of chronic hepatitis C
Source: BMC Med Genomics. 2010 Oct 22;3:48. doi: 10.1186/1755-8794-3-48 (PMC2984584; doi:10.1186/1755-8794-3-48)
Supplement: Additional file 1 — miRNA hypothetical target genes according to in silico analysis. [file 1755-8794-3-48-S1.PDF]

additional file 1. miRNA hypothetical target genes according to in silico analysis

Accession No.	Description	Gene symbol	target search algorithm		
			**PicTar**	**miRANDA/ miRNA Viewer**	**TargetScanS**
NM_013229.2	apoptotic peptidase activating factor 1	APAF1	miR-27b	miR-27b	
NM_001188.3	BCL2-antagonist/killer 1	BAK1		miR-27b	
NM_012342.2	BMP and activin membrane-bound inhibitor homolog	BAMBI		miR-18	
NM_138761.3	BCL2-associated X protein	BAX		miR-34b	
NM_138576.2	B-cell CLL/lymphoma 11B	BCL11B			miR-34
NM_000633.2	B-cell CLL/lymphoma 2	BCL2	miR-34b, miR-143	miR-34b	miR-34, miR-143,
NM_138578.1	BCL2-like 1	BCL2L1		miR-34b	
NM_138621.3	BCL2-like 11	BCL2L11		miR-24, miR-34b	
NM_005178.4	B-cell CLL/lymphoma 3	BCL3	miR-27b	miR-27b	
NM_001706.3	B-cell CLL/lymphoma 6	BCL6			miR-34
NM_001123383.1	BCL6 co-repressor	BCOR	miR-27b		
NM_001167.2	X-linked inhibitor of apoptosis	BIRC4		miR-143	miR-27
NM_001204.6	bone morphogenetic protein receptor, type II	BMPR2	miR-27b, miR-145		
NM_015981.3	calcium/calmodulin-dependent protein kinase II alpha	CAMK2A	miR-27b	miR-145, miR-34b	miR-27
NM_199141.1	coactivator-associated arginine methyltransferase 1	CARM1		miR-378	
NM_032982.2	caspase 2	CASP2			miR-34
NM_033340.2	caspase 7	CASP7		miR-18	
NM_005188.2	Cas-Br-M (murine) ecotropic retroviral transforming sequence	CBL		miR-18	
NM_002988.2	chemokine (C-C motif) ligand 18	CCL18		miR-27b	
NM_004591.2	chemokine (C-C motif) ligand 20	CCL20		miR-145	
NM_006139	CD28 molecule	CD28	miR-27b	miR-27b	
NM_000616	CD4 molecule	CD4		miR-378	
NM_004379.3	cAMP responsive element binding protein 1	CREB1	miR-34b, miR-27b	miR-34b	miR-27

NM_004380.2	CREB binding protein	CREBBP		miR-145	
NM_006565	CCCTC-binding factor	CTCF		miR-27b	
NM_005214.3	cytotoxic T-lymphocyte-associated protein 4	CTLA4		miR-145	
NM_018947.4	cytochrome c, somatic	CYCS		miR-34b, miR-378	
NM_175629.1	DNA (cytosine-5-)-methyltransferase 3 alpha	DNMT3A	miR-143	miR-143	
NM_032482.2	DOT1-like, histone H3 methyltransferase	DOT1L	miR-27b	miR-27b	miR-27
NM_004419.3	dual specificity phosphatase 5	DUSP5	miR-27b	miR-27b, miR-18	
NM_001946.2	dual specificity phosphatase 6	DUSP6		miR-145	miR-145
NM_005225.2	E2F transcription factor 1	E2F1		miR-18	
NM_024007.3	early B-cell factor 1	EBF1		miR-18	
NM_012199	eukaryotic translation initiation factor 2C, 1	EIF2C1	miR-145	miR-145, miR-378	
NM_012154.2	eukaryotic translation initiation factor 2C, 2	EIF2C2	miR-27b	miR-27b	miR-27
NM_017629	eukaryotic translation initiation factor 2C, 4	EIF2C4			miR-34
NM_198241.1	eukaryotic translation initiation factor 4 gamma, 1	EIF4G1	miR-34b	miR-378, miR-34b	
NM_001419.2	ELAV (embryonic lethal, abnormal vision, Drosophila)-like 1	ELAVL1		miR-34b	
NM_004432	ELAV (embryonic lethal, abnormal vision, Drosophila)-like 2	ELAVL2		miR-34b, miR-27b	
NM_005229.3	ELK1, member of ETS oncogene family	ELK1		miR-143	
NM_003824.3	Fas (TNFRSF6)-associated via death domain	FADD		miR-27b	
NM_004001.3	Fc fragment of IgG, low affinity IIb, receptor (CD32)	FCGR2B		miR-34b,	
NM_032682.4	forkhead box P1	FOXP1	miR-34b	miR-34b	miR-34
NM_002037.3	FYN oncogene related to SRC, FGR, YES	FYN	miR-27b		miR-27
NM_001002295.1	GATA binding protein 3	GATA3	miR-27b	miR-34b	miR-27

NM_002086.4	growth factor receptor-bound protein 2	GRB2	miR-27b	miR-27b	
NM_015401.3	histone deacetylase 7	HDAC7A		miR-145	
NM_001530.3	hypoxia inducible factor 1, alpha subunit	HIF1A	miR-18	miR-18	miR-18
NM_005516.4	major histocompatibility complex, class I, E	HLA-E		miR-34b	
NM_021958.3	H2.0-like homeobox	HLX1	miR-27b		miR-27
NM_000619.2	interferon, gamma	IFNG		miR-378	
NM_000572.2	interleukin 10	IL10	miR-27b		miR-27
NM_170743.2	interleukin 28 receptor, alpha	IL28RA		miR-378	
NM_000417.1	interleukin 2 receptor, alpha	IL2RA		miR-378	
NM_000565.2	interleukin 6 receptor	IL6R		miR-18	
NM_002198.2	interferon regulatory factor 1	IRF1		miR-378	
NM_002199.3	interferon regulatory factor 2	IRF2	miR-18	miR-18	
NM_002460.2	interferon regulatory factor 4	IRF4	miR-27b		miR-27
NM_006147.2	interferon regulatory factor 6	IRF6	miR-34b	miR-34b	
NM_001572.3	interferon regulatory factor 7	IRF7	miR-145		
NM_025194.2	inositol 1,4,5-trisphosphate 3-kinase C	ITPKC	miR-27b	miR-27b	
NM_003685.2	KH-type splicing regulatory protein	KHSRP	miR-27b		miR-27
NM_033360.2	v-Ki-ras2 Kirsten rat sarcoma viral oncogene homolog	KRAS	miR-27b, miR-143, miR-145		miR-143, miR-27
NM_002317.4	lysyl oxidase	LOX	miR-145		miR-145
NM_206943.1	latent transforming growth factor beta binding protein 1	LTBP1		miR-18	
NM_002755	mitogen-activated protein kinase kinase 1	MAP2K1		miR-34b	
NM_003954.2	mitogen-activated protein kinase kinase kinase 14	MAP3K14	miR-27b		miR-27
NM_003188.2	mitogen-activated protein kinase kinase kinase 7	MAP3K7	miR-143		miR-143
NM_015093.3	mitogen-activated protein kinase kinase kinase 7 interacting protein 2	MAP3K7IP2	miR-27b		miR-27

NM_002745.4	mitogen-activated protein kinase 1	MAPK1	miR-34b, miR-145	miR-378	
NM_002753.2	mitogen-activated protein kinase 10	MAPK10		miR-34b	
NM_001315.2	mitogen-activated protein kinase 14	MAPK14	miR-27b	miR-27b, miR-378	
NM_139033.2	mitogen-activated protein kinase 7	MAPK7			miR-27,
NM_018328	methyl-CpG binding domain protein 5	MBD2		miR-27b	miR-145
NM_004992.3	methyl CpG binding protein 2	MECP2	miR-34b, miR-18a, miR-145	miR-378, miR-18	miR-34
NM_005933.2	myeloid/lymphoid or mixed-lineage leukemia	MLL		miR-34b, miR-378, miR-27b	
NM_004995.2	matrix metallopeptidase 14	MMP14		miR-378	
NM_002467.3	v-myc myelocytomatosis viral oncogene homolo	MYC	miR-34b	miR-34b	
NM_000615.5	neural cell adhesion molecule 1	NCAM		miR-27b	
NM_003743.4	nuclear receptor coactivator 1	NCOA1		miR-378	miR-18
NM_181659.1	nuclear receptor coactivator 3	NCOA3		miR-18	
NM_006311.2	nuclear receptor co-repressor 1	NCOR1		miR-34b	
NM_002505.4	nuclear transcription factor Y, alpha	NF-YA		miR-378	
NM_138714.2	nuclear factor of activated T-cells	NFAT5	miR-34b, miR-18a	miR-27b	
NM_172390.1	nuclear factor of activated T-cells, cytoplasmic, calcineurin-dependent 1	NFATC1		miR-18	
NM_033004.3	NLR family, pyrin domain containing 1	NLRP1		miR-143	miR-143
NM_006092.2	nucleotide-binding oligomerization domain containing 1	NOD1		miR-27b	
NM_017617.3	Notch homolog 1, translocation-associated	NOTCH1	miR-34b		miR-34
NM_003884.4	K(lysine) acetyltransferase 2B	PCAF	miR-27b		
NM_016166.1	protein inhibitor of activated STAT, 1	PIAS1	miR-34b	miR-34b	
NM_006099.3	protein inhibitor of activated STAT, 3	PIAS3	miR-18a		miR-18
NM_000937.3	polymerase (RNA) II (DNA directed) polypeptide A, 220kDa	POLR2A		miR-34b	
NM_138712.3	peroxisome proliferator-activated receptor gamma	PPARG	miR-27b	miR-27b	miR-27
NM_000945.3	protein phosphatase 3 (formerly 2B), regulatory subunit B, alpha isoform	PPP3R1	miR-27b	miR-378	miR-27

NM_001198.3	PR domain containing 1, with ZNF domain	PRDM1			miR-145
NM_006254.3	protein kinase C, delta	PRKCD	miR-27b		miR-27
NM_005400.2	protein kinase C, epsilon	PRKCE	miR-143	miR-143	miR-143
NM_000448.2	recombination activating gene 1	RAG1		miR-378	
NM_000964.2	retinoic acid receptor, alpha	RARA	miR-27b	miR-145, miR-27b, miR-18	miR-27
NM_000965.3	retinoic acid receptor, beta	RARB			miR-34
NM_000321.2	retinoblastoma 1	RB1	miR-143		
NM_002919.2	regulatory factor X, 3	RFX3	miR-27b		miR-27
NM_032491.4	regulatory factor X, 4	RFX4		miR-145	
NM_000538.3	regulatory factor X-associated protein	RFXAP		miR-27b	
NM_006871.3	receptor-interacting serine-threonine kinase 3	RIPK3		miR-378, miR-18	
NM_012238.4	sirtuin (silent mating type information regulation 2 homolog) 1	SIRT1			miR-34
NM_005901.4	SMAD family member 2	SMAD2	miR-18a		miR-18
NM_005902.3	SMAD family member 3	SMAD3		miR-18	
NM_001001419.1	SMAD family member 5	SMAD5		miR-145, miR-27b	
NM_003069.3	SWI/SNF related, matrix associated, actin dependent regulator of chromatin, subfamily a, member 1	SMARCA1	miR-27b	miR-27b	
NM_003074.3	SWI/SNF related, matrix associated, actin dependent regulator of chromatin, subfamily c, member 1	SMARCC1		miR-34b	
NM_003076.4	SWI/SNF related, matrix associated, actin dependent regulator of chromatin, subfamily d, member 1	SMARCD1	miR-145,		
NM_001098426.1	SWI/SNF related, matrix associated, actin dependent regulator of chromatin, subfamily d, member 2	SMARCD2	miR-143	miR-143	
NM_022739.3	SMAD specific E3 ubiquitin protein ligase 2	SMURF2		miR-27b	
NM_003955.3	suppressor of cytokine signaling 3	SOCS3		miR-378	

NM_199421.1	suppressor of cytokine signaling 4	SOCS4	miR-27b	miR-378	miR-34, miR-27
NM_014011.4	suppressor of cytokine signaling 5	SOCS5	miR-18a	miR-18, miR-34b	
NM_004232.3	suppressor of cytokine signaling 6	SOCS6	miR-27b,	miR-27b	
NM_014598.1	suppressor of cytokine signaling 7	SOCS7			miR-145
NM_001080547.1	spleen focus forming virus (SFFV) proviral integration oncogene spi1	SPI1		miR-378	
NM_003473.2	signal transducing adaptor molecule (SH3 domain and ITAM motif) 1	STAM		miR-145	
NM_005819.4	syntaxin 6	STX6		miR-378	miR-27
NM_003173.2	suppressor of variegation 3-9 homolog 1	SUV39H1		miR-18	
NM_005638.4	vesicle-associated membrane protein 7	SYBL1			miR-143
NM_024665.4	transducin (beta)-like 1 X-linked receptor 1	TBL1XR1			miR-34
NM_021025.2	T-cell leukemia homeobox 3	TLX3		miR-18	
NM_003842.4	tumor necrosis factor receptor superfamily, member 10b	TNFRSF10B		miR-34b	
NM_005658.3	TNF receptor-associated factor 1	TRAF1		miR-145	
NM_004619.3	TNF receptor-associated factor 5	TRAF5		miR-18	
NM_014232.2	vesicle-associated membrane protein 2	VAMP2	miR-34b		
NM_004781.3	vesicle-associated membrane protein 3	VAMP3	miR-34b	miR-34b	
NM_020750.2	exportin 5	XPO5		miR-34b	
NM_004559.3	Y box binding protein 1	YBX1		miR-18,	
NM_003407.2	zinc finger protein 36, C3H type, homolog	ZFP36	miR-27b	miR-27b	miR-27
NM_007324.2	zinc finger, FYVE domain containing 9	ZFYVE9	miR-145	miR-145	miR-145
