# Supplementary figures and images for: Hepatic microRNA expression is associated with the response to interferon treatment of chronic hepatitis C
Source: BMC Med Genomics. 2010 Oct 22;3:48. doi: 10.1186/1755-8794-3-48 (PMC2984584; doi:10.1186/1755-8794-3-48)

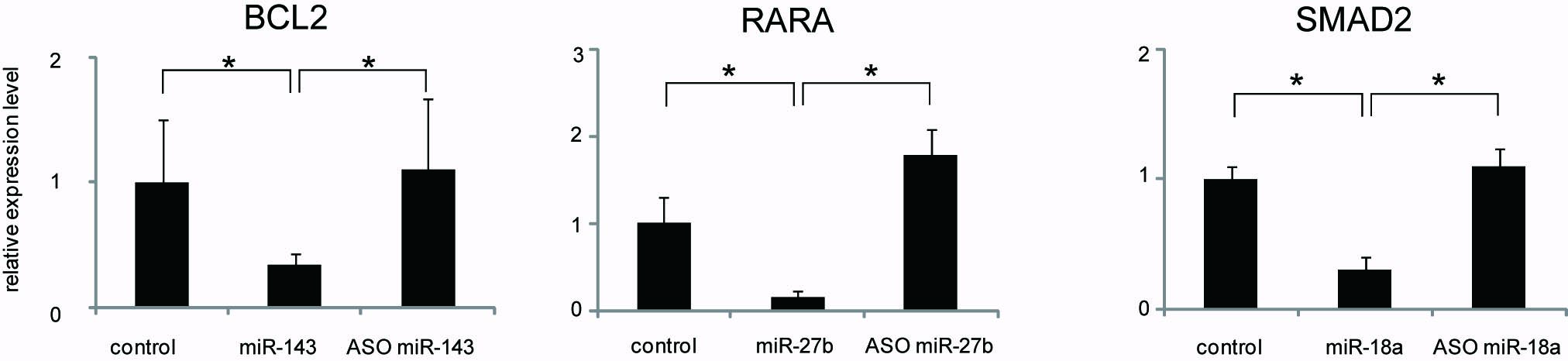

Supplement: Additional file 2 — Real-time qPCR validation of immune-related hypothetical target genes of miRNAs. The expression levels of hypothetical target genes in HEK293 cells were compared among three groups treated with control RNA, ds miRNA, and ASO miRNA. The data shown are means+SD of three independent experiments. Asterisk indicates a significant difference of p < 0.05. [file 1755-8794-3-48-S2.JPEG]
