# Supplementary material for: Hepatic microRNA expression is associated with the response to interferon treatment of chronic hepatitis C
Source: BMC Med Genomics. 2010 Oct 22;3:48. doi: 10.1186/1755-8794-3-48 (PMC2984584; doi:10.1186/1755-8794-3-48)
Supplement: Additional file 3 — human miRNA target on the HCV genome genotype 1b (Accession No. AF333324) [file 1755-8794-3-48-S3.PDF]

additional file 3. human miRNA target on the HCV genome genotype 1b (Accession No.AF333324)

<table>
<tr><th></th><th>gene</th><th>Target location and orientation</th><th>Hybridization</th><th>miRanda</th><th>TargetScan</th></tr>
<tr><td>NR vs. SVR</td><td>has-miR-34b</td><td>23-1</td><td>GUUAGUC-GAUUACUGUGACGGAu</td><td>yes</td><td>yes</td></tr>
<tr><td></td><td></td><td></td><td>|:| :| || :||::|||||||</td><td></td><td></td></tr>
<tr><td></td><td>HCV</td><td>272 - 295</td><td>CGAAAGGCCTTGTGGTACTGCCTg</td><td></td><td></td></tr>
<tr><td></td><td>has-miR-145</td><td>23-1</td><td>uucCCUAAGGACC-CUU-UUGACCUg</td><td>yes</td><td>no</td></tr>
<tr><td></td><td></td><td></td><td> | :||||||| ||| | |||||</td><td></td><td></td></tr>
<tr><td></td><td>HCV</td><td>8199 - 8224</td><td>gtcGAGTTCCTGGTGAATACCTGGAa</td><td></td><td></td></tr>
<tr><td></td><td>has-miR-145</td><td>23-1</td><td>uucccUAAGG-AC--CCUUUUGACCUG</td><td>yes</td><td>no</td></tr>
<tr><td></td><td></td><td></td><td> ||||| || ||::||||||:|</td><td></td><td></td></tr>
<tr><td></td><td>HCV</td><td>1421 - 1447</td><td>ctactATTCCATGGTGGGGAACTGGGC</td><td></td><td></td></tr>
<tr><td></td><td>has-miR-145</td><td>23-1</td><td>UUCCCUAAGGACCCUUUUGACCUg</td><td>yes</td><td>no</td></tr>
<tr><td></td><td></td><td></td><td>||||||| ::||| :||||||</td><td></td><td></td></tr>
<tr><td></td><td>HCV</td><td>6272 - 6292</td><td>AAGGGATGTTTGG---GACTGGAt</td><td></td><td></td></tr>
<tr><td></td><td>has-miR-18a</td><td>22-1</td><td>AUA--GA-CGUGAUCUACGUGGaau</td><td>yes</td><td>no</td></tr>
<tr><td></td><td></td><td></td><td>||| || ||||| |||||||</td><td></td><td></td></tr>
<tr><td></td><td>HCV</td><td>1794 - 1816</td><td>TATTGCTGGCACT--ATGCACCcca</td><td></td><td></td></tr>
<tr><td></td><td>has-miR-18a</td><td>22-1</td><td>auagacGUG-AUCUACGUG-GAAu</td><td>yes</td><td>no</td></tr>
</table>

```
                                              ||| ||||||||| |||
               HCV            5034 - 5057     ctcaccCACATAGATGCACACTTc

               has-miR-18a    22-1            auaGACGUG-AUC--UACGUGGAAu       yes       no
                                                 || ||| |:|  | ||:||||
               HCV            7604 - 7628     gtcCTACACATGGACAGGCGCCTTg

               has-miR-27b    21-1            CGUCUUGAA------UCGGUGACACUu     yes       yes
                                              | ||:||||      || ||||||||
               HCV            7967 - 7993     GAAGGACTTGCTGGAAGACACTGTGAc

               has-miR-378    20-1            CCGGAAGACUGAGG-------UUCAGGUc   yes       no
                                              || || ||| ||||      :||||||
               HCV            5573 - 5599     GG-CTGCTG-CTCCCGTGGTGGAGTCCAa

               has-miR-378    20-1            ccGGAA---GACUGAGGUUCAGGuc       yes       no
                                                ||||   | |||| |||||||
               HCV            7754 - 7778     caCCTTTGACAGACTGCAAGTCCtg


R      vs.
SVR            has-let-7a     22-1            UUGAUAUGUUGGAUGA-UGGAGu         yes       no
                                              |:||:| | |||| || |||||
               HCV            1207 - 1228     AGCTGTTC-ACCTTCTCACCTCg
```

has-let-7a	22-1	uuGAUAUGUUGGAUGAUGGAGU	yes	no
		`  | | | ::: ||||||||||`		
HCV	8730 - 8751	ggCAAAAGGGTGTACTACCTCA		
has-let-7a	22-1	uUGAUAUGUUGGAU--GAUGGAGU	yes	no
		` |:|: :| ||:|:  ||||||||`		
HCV	8957 - 8980	cATTGAGCCACTTGACCTACCTCA		
has-let-7f	22-1	uuGAUAUGUUAGAUGAUGGAGU	yes	no
		`  | | | ::| ||||||||||`		
HCV	8730 - 8751	ggCAAAAGGGTGTACTACCTCA		
has-let-7g	21-1	ugacauguuugAUGAUGGAGU	yes	no
		`           ||||||||||`		
HCV	8731 - 8751	gcaaaagggtgTACTACCTCA		
has-miR-100	22-1	guGUUCAAGCCUAGAUGCCCaa	yes	no
		`  ||||||:||  |||:|||`		
HCV	7893 - 7912	tcCAAGTTTGG--CTATGGGgc		
has-miR-100	22-1	guguucaaGCCUAGAUGCCCaa	yes	no
		`        | ||||||||||`		
HCV	8926 - 8947	tggactgcCAGATCTACGGGgc		

```
has-miR-10a    23-1           GUGUUUAAGCCUAGAUGUCCCAu        yes    no
                              | | |: |  |||| |:|||||
HCV            9031 - 9053    CTCCAGGTGAGATCAATAGGGTg

has-miR-126    21-1           CGUAAUAAUGAGUGCCAUGCu          yes    no
                              ||||     | | ||||:||
HCV            5839 - 5853    GCAT-----CGC-CGGTGCGg

has-miR-126    21-1           cGUAAUAAU-GAGU-GCCAUGCU        yes    no
                               | || | | |||: |||||:||
HCV            1215 - 1237    aCCTTCTCACCTCGCCGGTATGA

has-miR-126    21-1           cGUAAUAAUG-AGU---GCCAUGCu      yes    no
                               |||  | || |||   ||||:||
HCV            3746 - 3770    aCATGCTGACGTCATTCCGGTGCGc

has-miR-370    21-1           gGUCCAAGGUGGGGUCGUCCg          yes    no
                               ||| |  ||| :|| ||||
HCV            1594 - 1614    gCAGCTGGCACATCAACAGGa

has-miR-370    21-1           GGUCCAAGGUGGG--GUCGUCCG        yes    no
                              ||: || :::||:  || |||||
HCV            5353 - 5375    CCGCGTATTGCCTGACAACAGGC
```

has-miR-370	21-1	ggucсAAGGUG--GGGUCGUCcg	yes	no
		| |||| | ||||||		
HCV	6871 - 6893	accccTCCCACATCACAGCAGaa		
has-miR-370	21-1	gGUCCAAG----G--UGGGGUCGUCCg	yes	no
		| ||||| | ::: ||||:||		
HCV	9244 - 9270	gCTGGTTCGTTGCTGGTTACAGCGGGg		
has-miR-370	21-1	GGUCCAAGGUGG----GGUC-GUCCG	yes	no
		|: || || || |||| |||||		
HCV	8330 - 8353	CTTGG--CCCCCGAAGCCAGACAGGC		
has-miR-370	21-1	GGUCCAAG-GUGGG-----GUCGUCcg	yes	no
		||| | || |: || ||||||		
HCV	1072 - 1098	CCACGCTCGCGGCCAGGAACAGCAGca		
has-miR-370	21-1	GGUCCAAG-GUGG--GGUCGUCCG	yes	no
		||||| | |::| |::||||||		
HCV	3656 - 3679	CCAGGACCTCGTCGGCTGGCAGGC		
has-miR-370	21-1	GGUCCAAGGUGGG--------GUCGUCCg	yes	no
		| ||| ||| || |:|||||		
HCV	7022 - 7048	CGAGG--CCAACCTCCTGTGGCGGCAGGa		

<table>
<tr><td>has-miR-370</td><td>21-1</td><td>GGUCCAAGGUGGGGUCG-UCCG</td><td>yes</td><td>no</td></tr>
<tr><td></td><td></td><td>|| || || :||||| ||||</td><td></td><td></td></tr>
<tr><td>HCV</td><td>7922 - 7942</td><td>CC-GGAACCTATCCAGCAAGGC</td><td></td><td></td></tr>
<tr><td>has-miR-370</td><td>21-1</td><td>ggucCAAGGUGGGG-UCGUCCG</td><td>yes</td><td>no</td></tr>
<tr><td></td><td></td><td> || ||| || |||||||</td><td></td><td></td></tr>
<tr><td>HCV</td><td>5056 - 5077</td><td>tcttGTCCCAGACCAAGCAGGC</td><td></td><td></td></tr>
<tr><td>has-miR-370</td><td>21-1</td><td>gGUCCAAGGU--GGGGUCGUCCG</td><td>yes</td><td>no</td></tr>
<tr><td></td><td></td><td> |:|| | || |::|||||||</td><td></td><td></td></tr>
<tr><td>HCV</td><td>5650 - 5672</td><td>gCGGGATACAGTACTTAGCAGGC</td><td></td><td></td></tr>
<tr><td>has-miR-370</td><td>21-1</td><td>GGUCCAAGGUGGGGUCGUCCg</td><td>yes</td><td>no</td></tr>
<tr><td></td><td></td><td>|| || | |::|||||| ||</td><td></td><td></td></tr>
<tr><td>HCV</td><td>9215 - 9235</td><td>CCCGGCTGCGTCCCAGCTGGa</td><td></td><td></td></tr>
<tr><td>has-miR-98</td><td>22-1</td><td>uUGUUAUGUUGAA--UGAUGGAgu</td><td>yes</td><td>no</td></tr>
<tr><td></td><td></td><td> | :| ||||||| ||||||</td><td></td><td></td></tr>
<tr><td>HCV</td><td>9215 - 9235</td><td>cAGGAGACAACTTCCCCTACCTgg</td><td></td><td></td></tr>
<tr><td>has-miR-98</td><td>22-1</td><td>uuGUUA--UGUUGAAU--GAUGGAGU</td><td>yes</td><td>no</td></tr>
<tr><td></td><td></td><td> || | :| ||||: ||||||||</td><td></td><td></td></tr>
<tr><td>HCV</td><td>9215 - 9235</td><td>tcCATTGAGCCACTTGACCTACCTCA</td><td></td><td></td></tr>
</table>

<table>
<tr><td>has-miR-98</td><td>22-1</td><td>uuGUUAUGUUGAAUGAUGGAGU</td><td>yes</td><td>no</td></tr>
<tr><td></td><td></td><td> ||| | ::: ||||||||||</td><td></td><td></td></tr>
<tr><td>HCV</td><td>9215 - 9235</td><td>ggCAAAAGGGTGTACTACCTCA</td><td></td><td></td></tr>
</table>

Abbreviations: Vertical bars indicated the complementary bases between the HCV replicon genome and miRNA.

The G:U or T:G wobble pair also was shown as colon. Numbers of HCV sequences of 5'–UTR show nucleotide from 5' end.
